# Long non-coding RNA TUG1 knockdown prevents neurons from death to alleviate acute spinal cord injury via the microRNA-338/BIK axis

**DOI:** 10.1080/21655979.2021.1966258

**Published:** 2021-09-14

**Authors:** Hongbo Wu, Yi Li, Xiaofeng Wang, Zhiwen Zhang, Yuliang Huang

**Affiliations:** Department of Orthopaedics, Huizhou City Center People’s Hospital, Huizhou Guangdong, P.R. China

**Keywords:** Long non-coding rna tug1, microRNA-338, bik, spinal cord injury

## Abstract

Taurine up-regulated gene 1 (TUG1) is a cancer-associated long noncoding RNA (lncRNA) and engages in the development of spinal cord injury (SCI), a suffering neuropathological disorder. However, the regulatory role of TUG1 in acute SCI (ASCI) is still underdetermined. RT-qPCR and western blot analysis were applied to measure the expression of TUG1, microRNA-338 (miR-338), Bcl2-interacting killer (BIK), cleaved caspase 3 (c-caspase 3) and hypoxia-inducible factor-1 alpha (HIF-1α) in ASCI rats and hypoxic cells. Cell death was evaluated using flow cytometric analysis. The relationships among miR-338, TUG1 or BIK were confirmed by luciferase reporter assay, RNA immunoprecipitation and RNA pull-down. Accordingly, we monitored higher expression of TUG1 and BIK, but lower expression of miR-338 in ASCI rats and hypoxic cells. *In vitro*, hypoxia expedited cell death and c-caspase 3 levels. *In vivo*, ASCI rats were successfully developed as evidenced by diminished Basso-Beattie-Bresnahan (BBB) locomotor score and enhanced c-caspase 3 and HIF-1α expression. Nevertheless, TUG1 knockdown mitigated the cell death in ASCI rats and hypoxic cells. Mechanically, TUG1 interacted with miR-338 to regulate the BIK expression. Together, TUG1 silencing could alleviate the death in neurons and ASCI models via modulating the miR-338/BIK axis.

## Graphical abstract

**Figure:**
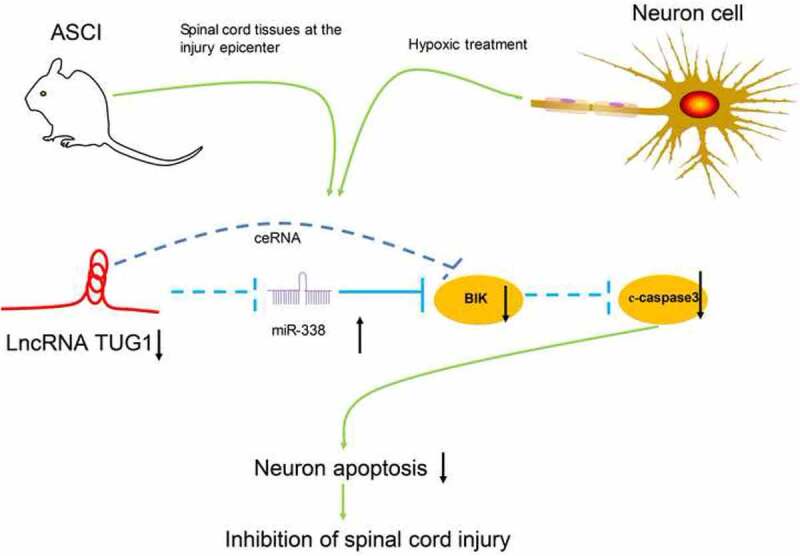

## Introduction

Spinal cord injury (SCI) is a neurological disorder, causing great socioeconomic impacts on sufferers and the health care system [1]. The traumatic causes involve fractures, car accidents, work-related falls, violence as well as sports activities, while the non-traumatic causes include ischemia, infection, along with cancers [2]. Existing clinical strategic options are comprised of intensive care measures, surgical decompression at early stage, stabilization and blood pressure augmentation to decrease secondary injury [3]. Moreover, acute central nervous system injuries change the expression pattern of protein-coding genes that modulate many events, such as oxidative stress, inflammatory response as well as apoptosis that synergistically enhance neuronal death following an injury [4]. After SCI, permanent neuronal loss is a major challenge and may contribute to dysfunction, thereby promoting the survival of neurons is of great importance for recovery of patients with SCI [5].

Long non-coding RNAs (lncRNAs) are termed as non-protein-coding transcripts longer than 200 nucleotides, and several lncRNAs, involving metastasis-associated lung adenocarcinoma transcript 1 and X–inactive specific transcript have been acknowledged to involve in the modulation of SCI [6]. Declines in lncRNA taurine-upregulated gene 1 (TUG1) expression inhibited inflammatory response in rats with spinal cord ischemia reperfusion and SCI [7]. However, its relevance to neuronal death, another distinctive feature of SCI, remains unsettled. Interestingly, microRNAs (miRNAs) and lncRNAs could repress each other and perform as competing endogenous RNAs (ceRNAs), thus generating a lncRNAs-miRNAs-mRNAs network [8]. For instance, knockdown of NEAT1 relieved the inflammatory response of SCI through targeting miR-211-5p/MAPK1 axis [9]. The miRNA sponge role of lncRNA TUG1 has been highlighted in cerebral ischemia/reperfusion injury by positively mediating the miR-145/aquaporin-4 axis [10]. In the current work, miR-338 was observed to share a binding relationship with TUG1. Moreover, small nucleolar RNA host gene 15 was found to serve as a ceRNA to regulate miR-338-3p and FKBP1A expression in prostate cancer [11]. Besides, miR-338 was applied to enhance axonal regeneration following peripheral nerve injury [12]. Bcl2-interacting killer (BIK), a major member of the BH3-only Bcl-2 family, is an unfavorable prognostic marker for breast cancer [13]. According to the bioinformatic tool, miR-338 was predicted to share a binding relationship with BIK. It has been reported that spinal cord compression leads to a gradual decrease in blood flow, resulting in chronic hypoxia [14]. Even after a short (15–20 min) period of hypoxia, neural necrosis will rapidly ensue within 6 h, and damaged cells will continue to die from apoptosis for 24–48 h [15]. Therefore, motor function in spinal cord injured rats can be improved by reducing hypoxia [16]. Henceforth, we employed human neuronal cell line AGE1.HN and rat pheochromocytoma PC12 cells upon hypoxia *in vitro* and rat acute SCI (ASCI) models *in vivo* to determine our hypothesis whether lncRNA TUG1 regulated SCI progression and, if yes, whether miR-338/BIK takes part in the regulation of TUG1-mediated neuronal death. The aim and goal of this study was to probe the underlying mechanisms of lncRNA TUG1 suppression in the reduction of neuronal death through the miR-338/BIK axis in SCI rats.

## Materials and methods

### Establishment of a rat model

All the procedures regarding animals were permitted and monitored by the ethics committee of Huizhou City Center People’s Hospital (approval number: IACUC-2019-B81). Sprague-Dawley rats (200–250 g) obtained from Beijing Vital River Laboratory Animal Technology Co., Ltd. (Beijing, China) were bred under controlled temperature at 23–25°C with standard rodent food and unlimited water before the surgery in the Department of Orthopedics, Huizhou City Center People’s Hospital. The sample size was determined according to previous reports [17,18].

An ASCI animal model was established by contusion spinal cord injury as described previously [19]. Briefly, the rats were anaesthetized by an intraperitoneal injection of pentobarbital sodium at 50 mg/kg. The animals were incised along the midline of the back to expose the T9-11 thoracic spinal vertebrae under aseptic conditions. Subsequently, spinal cord compression was performed by striking T10 segments with a 10 g hammer from 25 mm high. A successful ASCI model induction was defined as lower limb paralysis. Sham-operated rats (n = 5) were treated the same as the ASCI-treated rats except for no contusion.

After the operation, all animals were treated with penicillin and analgesics for 3 days, and the bladder was manually emptied three times a day until the bladder function recovered in these rats. Within three hours post-operation, 25 ASCI rats were randomly divided into five groups (n = 5 per group) and subjected to intrathecal injections of 300 ug siRNA-negative control (NC), siRNA-TUG1, siRNA-TUG1 + NC or siRNA-TUG1 + miR-338 inhibitor or without any other injection via Entranster^TM^-in vivo transfection reagents (Engreen Biosystem Co., Ltd., Beijing, China) [20,21]. The rats requiring injection of two plasmids were administrated with the second injection 48 h after the first plasmid was injected.

The neurological function was evaluated by Basso, Beattie and Bresnahan (BBB) score 15 days following the surgery. Afterward, the rats were euthanized with excess pentobarbital sodium at 200 mg/kg, and T10 spinal cord tissues from the injury center were isolated for reverse transcription-quantitative polymerase chain reaction (RT-qPCR) as well as western blot.

### Functional analysis

After 15 days, locomotor activity of the rats was assessed by 21-point BBB scores, where 0 indicates no locomotor activity, while 21 indicates normal locomotor function [22]. During the assessment, three observers who did not know the grouping situation analyzed the hindlimb locomotor activity, stability, coordination, stepping, trunk position, toe gap, foot position and tail position of the rats, respectively, to obtain the average.

### Cell culture, treatment and transfection

Human neuronal cell line AGE1.HN and rat pheochromocytoma PC12 cells from the ATCC (Manassas, VA, USA) were cultivated in a 37°C incubator with 5% CO_2_ and Roswell Park Memorial Institute 1640 (Gibco, Carlsbad, CA, USA) containing 10% fetal bovine serum (Gibco) plus 100 μg/mL penicillin/streptomycin (Life Technology, Carlsbad, CA, USA). A SCI *in vitro* model was generated by exposing AGE1.HN and PC12 cells under a condition containing 3% O_2_, 5% CO_2_ and 92% N_2_ at 37°C for 24 h. AGE1.HN and PC12 cells cultured under normoxic conditions were utilized as controls.

Cells exposed to hypoxia were transfected to study the effect of gene expression alteration on SCI *in vitro*. Human or rat TUG1 and BIK sequences were synthesized and subcloned into pcDNA3.1 vectors (Invitrogen Inc., Carlsbad, CA, USA) to generate pcDNA-TUG1 and pcDNA-BIK. Whereas small interfering RNA (siRNAs) targeting TUG1 and BIK (siRNA-TUG1 and siRNA-BIK), miR-338 mimic/inhibitor were designed and synthesized by GenePharma Corporation (Shanghai, China). The empty pcDNA3.1 vector (pcDNA), scrambled siRNA (si-NC), and the corresponding scrambled miRNA (NC) were used as negative controls (Sequence information of the siRNAs and inhibitor used is listed in Table 1).

**Table 1.** List of siRNA and inhibitor sequences

Gene	Sequence (5ʹ-3ʹ)
siRNAs	
siRNA-TUG1 (hsa)	GGUUAUUGUUUAUAAUUUUUU
siRNA-TUG1 (rno)	GGGTTACTCAGGAACCAAAAC
siRNA-BIK (hsa)	GCCGCCGCCAGAGGAGAAAUG
siRNA-BIK (rno)	GUCUGGGCCGAGAGGUGUUCG
miRNA inhibitors	
miR-338 inhibitor (hsa)	AGGUCGUAGUCACUAAAACAAC
miR-338 inhibitor (rno)	AGGUCGUAGUCACUAAAACAACU

**Note**: siRNA, small interfering RNA; TUG1, taurine up-regulated gene 1; BIK, BCL2 interacting killer; miR-338, microRNA-338; hsa, homo sapiens; rno, rattus norvegicus.

Lipofectamine 2000 reagent (Invitrogen) was applied for all transfection according to the manufacturer’s instructions. Briefly, the cells were seeded into six-well plates at 5 × 10^4^ cells/well. At 60%-70% cell confluence, the complete medium was replaced with 1.9 mL serum-free medium. A total of 50 μL Opti-MEM serum-free medium (51,985,042, Gibco, Gaithersburg, MD, USA) was mixed gently with RNA 100 pmol/DNA 2 μg and allowed to stand at room temperature for 5 min. Lipofectamine 2000 (5 µL) was then mixed gently with 50 µL serum-free Opti-MEM and allowed to stand at room temperature for 5 min. The diluted lipofectamine 2000 was added to the diluted plasmids, incubated at room temperature for 20 min and then added dropwise to the 6-well plates. After a 4–6 h of culture, the serum-free medium was replaced with complete medium to continue the culture process. Transfection efficiency was measured after 48 h, and subsequent experiments were carried out.

### RT-qPCR

RT-qPCR was used to detect mRNA expression in tissues or cells [23]. Tissues or AGE1.HN and PC12 cells were collected for total RNA extraction using Trizol reagents (Invitrogen Inc). The extracted RNA was reversely transcribed into cDNA using a SuperScript reverse transcript kit (Invitrogen). The amplification of cDNA was performed with an ABI PRISM 7300 RT-PCR system (Applied Biosystems, Inc., Foster City, CA, USA). The calculation was carried out using the 2^−ΔΔCT^ method. Glyceraldehyde-3-phosphate dehydrogenase (GAPDH) or U6 were used as internal controls for TUG1/BIK or miRNA expression of their respective species. The primer sequences are listed in Table 2.

**Table 2.** Primer sequence used in this study

Species	Gene	Forward primer (5ʹ-3ʹ)	Reverse primer (5ʹ-3ʹ)	PCR Product (bp)	Temperate (°C)
Human	TUG1	CTGAAGAAAGGCAACATC	GTAGGCTACTACAGGATTTG	183	58
	miR-338	ATCCAGTGCGTGTCGTGG	TGCTTCCAGCATCAGTGAT	164	59
	BIK	CCCCGAGATAGTGCTGGAAC	GCCGAGGGCATCACATATCA	176	60
	GAPDH	GCACCGTCAAGGCTGAGAAC	TGGTGAAGACGCCAGTGGA	152	60
	U6	CTCGCTTCGGCAGCACAT	TTTGCGTGTCATCCTTGCG	163	59
Rat	TUG1	GACCTCAGACTGGTTGGGTG	TGCACTGGGTAAACGTTGGA	189	60
	miR-338	ACAATATCCTGGTGCTGAG	GAACATGTCTGCGTATCTC	174	58
	BIK	CTACGAATACTCGGGTGCGG	CACCTTCTGGGGTGTGAGAC	168	60
	GAPDH	CTCCTCGAAGTACCCTGTGC	CATGGTGCAGCGATGCTTTA	159	60
	U6	TGGAACGCTTCACGAATTTGCG	GGAACGATACAGAGAAGATTAGC	154	60

**Note**: TUG1, taurine-upregulated gene 1; miR-338, microRNA-338; BIK, Bcl2-interacting killer; GAPDH, glyceraldehyde-3-phosphate dehydrogenase.

### Western blot assays

Western blot experiments were used to detect protein expression in cells and in tissues [24]. The samples were lysed using radio-immunoprecipitation assay (RIPA) buffer (Beyotime, Shanghai, China) containing 50 mM Tris (pH = 7.4), 150 mM NaCl, 1% Triton X-100, 1% sodium deoxycholate, 0.1% sodium dodecyl sulfate (SDS), sodium orthovanadate, sodium fluoride, ethylenediaminetetraacetic acid and leupeptin and centrifuged at 4°C at 10,000 g for 20 min to remove insoluble fraction. Protein concentration was measured with a bicinchoninic acid protein concentration assay kit (Beyotime). Following SDS-PAGE, the separated proteins were transferred onto polyvinylidene fluoride membranes. The membranes were sealed for 1 h with a tris buffered saline with Tween-5% skim milk at room temperature and probed with 5% bovine serum albumin-diluted primary antibodies to HIF-1α (1:1000, ab179483, Abcam, Cambridge, UK), β-actin (1:2500, ab8227, Abcam), total caspase3 (1:2000, ab184787, Abcam), and c-caspase3 (1:5000, #9664, Cell Signaling Technologies, Beverly, MA, USA) overnight at 4°C. Subsequently, the membranes were probed with horseradish peroxidase-labeled secondary goat anti-rabbit antibody to IgG (1:50,000, ab205718, Abcam) at 20°C for 1.5 h. The blots were visualized with enhanced chemiluminescence (WBKLS0100, Millipore Corp, Billerica, MA, USA). The bands were quantified using Image Studio software (Li-Cor Biosciences, Lincoln, NE, USA). All antibodies used have been validated by the manufacturers for knockdown or widely used and have good specificity.

### Assessment of Annexin V/propidium iodide (PI) staining

Cell death was analyzed by flow cytometric analysis using the Annexin V-fluorescein isothiocyanate (FITC) death kit (BD Biosciences, San Jose, CA, USA) [25]. After hypoxia and transfection, AGE1.HN and PC12 cells (1 × 10^5^ cells/mL) were resuspended in binding buffer supplemented with Annexin V-FITC and PI at 4°C in the dark for 15 min. After that, the cells were loaded onto a flow cytometer (Beckman Coulter, Fullerton, CA, USA).

### Nuclear/Cytoplasmic fractionation

The nuclei and cytoplasm of AGE1.HN and PC12 cells were separated by PARIS kits (AM1921; Invitrogen) as previously described [26]. The expression of lncRNA TUG1 in the nucleus was determined, with U6 as the internal control of the nucleus and GAPDH as the positive internal of the cytoplasm.

### RNA immunoprecipitation (RIP)

RIP was used to verify the binding relationship of TUG1 and miR-338 [27]. After trypsin detachment, PC12 and AGE1.HN cells were lysed with RIPA buffer for 30 min at 4°C and centrifugated at 10,000 g for 20 min at 4°C to extract the supernatant. The A/G agarose beads were diluted with RIPA buffer until 50% concentration. The cell extracts were then ice-bathed with the primary antibodies to Ago2 (ab32381, Abcam) or IgG (ab125900 or ab181236, Abcam) coupled with A/G agarose beads. The particles were washed twice with RIPA buffer to remove nonspecific binding proteins. A portion of agarose beads-antigen antibody complexes was used to extract total RNA with Trizol reagents, and RT-qPCR was then conducted to examine the enrichment of miR-338 and TUG1. The remaining part was suspended with loading buffer containing 65 mM Tris-HCl (pH = 8.0), 10% (v/v) glycerol, 2.3% (w/v) SDS, 0.01% bromophenol blue and 1% dichlorodiphenyltrichloroethane. The sample was boiled for 5 min, centrifuged and subjected to electrophoresis, incubation with the secondary antibody and exposure.

### RNA pull-down

RNA pull-down was used to verify the binding relationship of lncRNA TUG1 and miR-338 [9]. The mixture of protein extracts from PC12 and AGE1.HN cells and 50 pmol biotinylated TUG1 was incubated for 1 h with 50 µL streptavidin agarose beads (Life Technologies) at 4°C or with biotinylated arbitrary single nucleotide as negative control (NC). The associated RNA-protein complexes were eluted with biotin elution buffer and boiled in SDS for 10 min. miR-338 expression was measured using RT-qPCR.

### Dual-luciferase reporter assay

Referring to a previous report [28], the binding relationship between miR-338 and BIK was verified by a dual-luciferase assay. Potential binding sites between miR-338 and BIK were predicted in StarBase (http://starbase.sysu.edu.cn/). BIK 3ʹuntranslated region (3ʹUTR) fragments containing miR-338 binding sites were cloned into PGL3 dual-luciferase reporter vectors (Promega, Madison, WI, USA), thus constructing wild-type luciferase vectors (WT-3ʹUTR). Mutant luciferase vectors (MT-3ʹUTR) were constructed by mutating BIK binding sites. The recombinant plasmids were co-transfected with miR-338 mimic/inhibitor or its negative control into AGE1.HN and PC12 cells for 24 h. After a 10-min treatment with RIPA (Beyotime) at room temperature, the luciferase activity was measured with a luciferase assay kit (Promega, Madison, WI, USA) as described by the manufacturer.

### Terminal deoxynucleotidyl transferase (TdT)-mediated dUTPbiotin nick end labeling (TUNEL)

TUNEL staining was used to detect cell death in the lesioned area of rat spinal cord [28]. After euthanasia, the heart was exposed by opening the abdominal cavity. A perfusion needle was then inserted at the left apical position along the aortic orifice, and the rat’s posterior vena cava was quickly clipped. First, the rats were perfused with 85% saline. When the effluent saline was blood-free, the rats were perfused with 4% paraformaldehyde. Immobilization was terminated when the rat’s limbs were stretched and stiffened and the liver, kidneys and lungs turned white. A cross-sectional section of the spinal cord injury was dissected, and the spinous processes and vertebral plates were opened to harvest the spinal cord tissues.

The spinal cord tissues were fixed with 4% paraformaldehyde at 4°C for 6 h, dehydrated with gradient alcohol (75%-100%), cleared with xylene and paraffin-embedded. Five spinal cord sections (3 μm) were obtained from each rat, and the TUNEL detection kit (YT137, Biolab, Beijing, China) was used. Paraffin-embedded sections were dewaxed with xylene and hydrated in gradient alcohol (100%-75%). After being washed with distilled water, the sections were soaked in 3% H_2_O_2_ for 10 min and detached with protease K (20 pg/mL) for 7 min. The samples were cultured with 20 μL staining solution at 37°C for 2 h and with 50 μL Tris-buffered saline-diluted streptavidin-biotin complex for 1 h at 37°C. The sections were then stained with diaminobenzidine for 10 min and washed with distilled water for 5 min. After being stained with hematoxylin for 1 min, the sections were sealed with neutral gum. Dead cells were characterized by dark brown staining of the nucleus and nuclear membrane. Three out of the five sections of the spinal cord from each rat were randomly selected for counting. Five randomly selected regions within each section were observed under an optical microscope (200 ×, CH30, Olympus Optical Co., Ltd., Tokyo, Japan). The rate of cell death was quantified by counting the percentage of dark brown stained cells versus blue stained cells using Image J.

### Statistics

The data were compared using SPSS 22.0 (IBM Corp. Armonk, N.Y., USA) statistical software. Data were shown in the form of mean ± standard deviation (SD). The normal distribution of the data was tested by the Shapiro-Wilk test, and the Levene’s test was used to detect variance homogeneity. One-way analysis of variance (ANOVA) was applied for the comparison of the measurement data between more than 2 groups followed by Tukey’s multiple comparisons test. Unpaired *t* test was utilized to compare 2 groups. The results of all *in vitro* experiments were the average of five independent experiments. Statistical significance was set at *p* < 0.05.

## Results

In this study, we employed human neuronal cell line AGE1.HN and rat pheochromocytoma PC12 cells upon hypoxia in vitro and rat ASCI models in vivo to determine our hypothesis whether lncRNA TUG1 regulated SCI progression and, if yes, whether miR-338/BIK takes part in the regulation of TUG1-mediated neuronal death. The aim and goal of this study was to probe the underlying mechanisms of lncRNA TUG1 suppression in the reduction of neuronal death through the miR-338/BIK axis. Flow cytometry, western blot and TUNEL staining were conducted to examine cell apoptosis, and nuclear/cytoplasmic fractionation, RIP, RNA pull-down, and dual-luciferase assay were conducted to measure the relationship among TUG1, miR-338, and BIK.

### LncRNA TUG1 is overexpressed in ASCI rats and hypoxic AGE1.HN and PC12 cells

**Figure 1.**
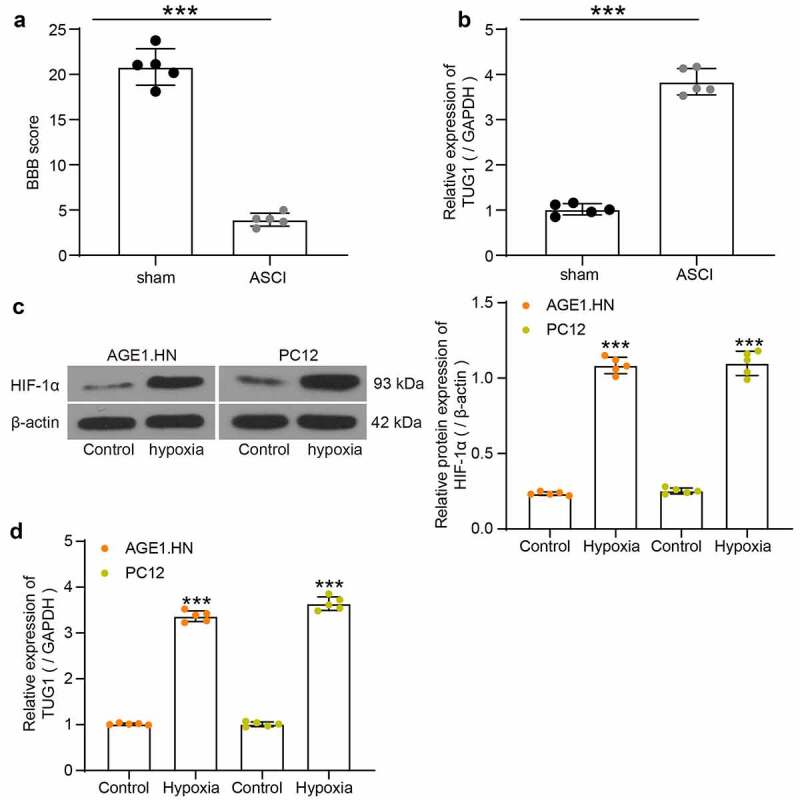
TUG1 expresses highly in SCI tissues and AGE1.HN and PC12 cells. Rats were categorized into sham group (n = 5) and ASCI group (n = 5). (a) BBB scores were evaluated (unpaired *t* test, ***p < 0.001; t(8) = 17.574, p < 0.001). (b) TUG1 expression in spinal cord tissues of ASCI and sham-operated rats determined by RT-qPCR normalized to GAPDH (unpaired *t* test, ***p < 0.001; t(5.414) = −19.860, p < 0.001). (c) the protein expression of HIF-1α in AGE1.HN and PC12 cells treated with and without hypoxia measured by western blot analysis normalized to β-actin (unpaired *t* test, ***p < 0.001, AGE1.HN: t(4.354) = −34.359, p < 0.001; PC12: t(4.453) = −22.790, p < 0.001). (d) TUG1 expression in AGE1.HN and PC12 cells treated with and without hypoxia measured by RT-qPCR normalized to GAPDH (unpaired *t* test, ***p < 0.001. AGE1.HN: t(4.215) = −44.328, p < 0.001; PC12: t(4.879) = −37.856, p < 0.001). Data were displayed as the mean ± SD. Five independent assays were conducted only for *in vitro* experiments

The hindlimb locomotor activities of five ASCI rats without other treatments and five sham-operated rats were assessed by BBB score after the rat modeling (Figure 1a). The BBB score of ASCI rats was decreased relative to the sham-operated rats. Sham-operated rats had consistent toe gap with paws placed parallel to the body during stance and could locomote steadily while keeping the tail up off the ground. While ASCI rats showed slight movements of hindlimb joints, indicating the successful establishment of the ASCI model. The spinal cord tissues from five ASCI rats without other treatments and from five sham-operated rats were collected to evaluate the lncRNA TUG1 expression using RT-qPCR. It was found that ASCI rats exhibited higher lncRNA TUG1 expression than that in the sham-operated rats (Figure 1b). Likewise, AGE1.HN and PC12 cells under hypoxia demonstrated elevated protein expression of HIF-1α relative to normoxic cells, proving the successful establishment of the hypoxia-treated cell model (Figure 1c). LncRNA TUG1 primers from human or rat were used for RT-qPCR to determine the expression of lncRNA TUG1 in AGE1.HN and PC12 cell. The expression of lncRNA TUG1 was increased in the AGE1.HN and PC12 cells after hypoxia treatment versus the cells without hypoxia treatment (Figure 1d).

### LncRNA TUG1 plays a role in promoting cell death in AGE1.HN and PC12 cells

**Figure 2.**
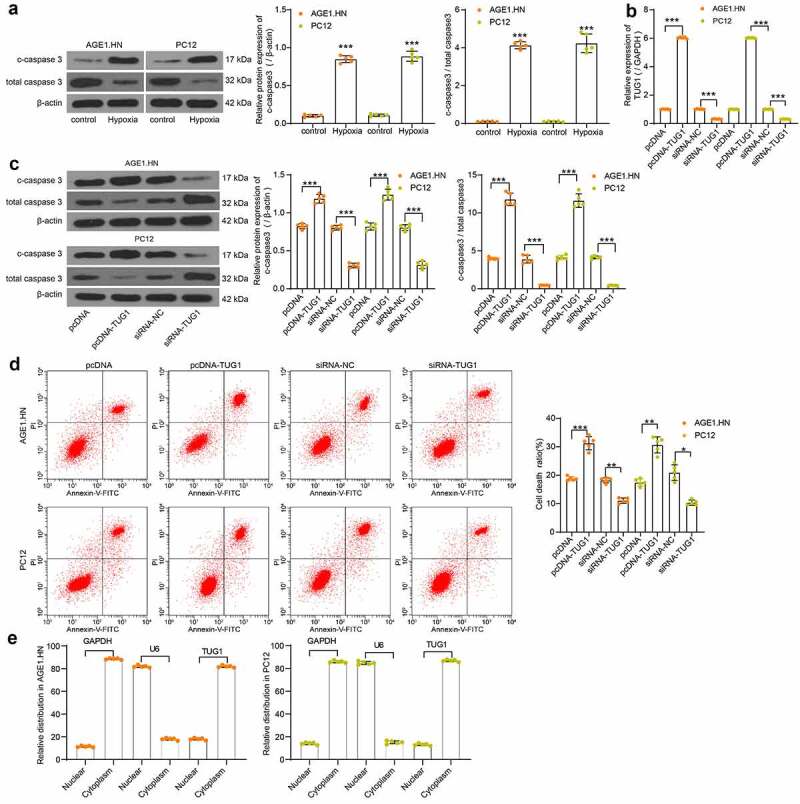
TUG1 promotes AGE1.HN and PC12 cell death. (a) the protein expression of c-caspase3 and c-caspase3/total caspase3 in AGE1.HN and PC12 cells treated with and without hypoxia measured by western blot analysis normalized to β-actin (unpaired *t* test, ***p < 0.001. For c-caspase3: AGE1.HN: t(5.061) = −35.105, p < 0.001; PC12: t(8) = −25.322, p < 0.001. For c-caspase3/total caspase3: AGE1.HN: t(8) = −49.07, p < 0.001; PC12: t(8) = −18.78, p < 0.001). Human or rat pcDNA-TUG1 and siRNA-TUG1 and their NCs were transfected into hypoxia-treated AGE1.HN and PC12 cells, respectively. (b) TUG1 expression in AGE1.HN and PC12 cells after transfection measured by RT-qPCR normalized to GAPDH (one-way ANOVA, ***p < 0.001. AGE1.HN: F(3,16) = 35,058.432, p < 0.001; PC12: F(3,16) = 70,046.402, p < 0.001). (c) the protein expression of c-caspase3 and c-caspase3/total caspase3 in AGE1.HN and PC12 cells after transfection measured by western blot analysis normalized to β-actin (one-way ANOVA, ***p < 0.001. For c-caspase3: AGE1.HN: F(3,16) = 416.825, p < 0.001; PC12: F(3,16) = 260.449, p < 0.001. For c-caspase3/total caspase3: AGE1.HN: F(3,16) = 475.2, p < 0.001; PC12: F(3,16) = 468.6, p < 0.001). (d) the death rate of AGE1.HN and PC12 cells after transfection measured by flow cytometry (one-way ANOVA, *p = 0.019, **p = 0.005, ***p < 0.001. AGE1.HN: F(3,16) = 184.670, p < 0.001; PC12: F(3,16) = 34.701, p < 0.001). (e) the distribution of TUG1 in AGE1.HN and PC12 cells evaluated by nuclear/cytoplasmic fractionation. Data were displayed as the mean ± SD. Five independent assays were conducted only for *in vitro* experiments

Western blot was subsequently performed to measure the c-caspase3 expression and c-caspase3/total caspase3 in the normoxic and hypoxic cells. We observed that the c-caspase3 expression and c-caspase3/total caspase3 was significantly enhanced after hypoxia treatment in AGE1.HN and PC12 cells (Figure 2a). Therefore, human or rat pcDNA-TUG1, siRNA-TUG1 and their NCs were transfected into hypoxia-treated AGE1.HN and PC12 cells. LncRNA TUG1 expression was then measured with RT-qPCR with their respective primers, and the transfection proved effective in AGE1.HN and PC12 cells (Figure 2b). Western blot analysis revealed that the administration of pcDNA-TUG1 promoted c-caspase3 expression and c-caspase3/total caspase3 in both AGE1.HN and PC12 cells, while siRNA TUG1 repressed the two levels (Figure 2c). Consistently, it was demonstrated by flow cytometry that pcDNA-TUG1 enhanced AGE1.HN and PC12 cell death, whereas siRNA TUG1 led to the opposite trends (Figure 2d). Subsequently, through the nuclear/cytoplasmic fractionation experiment, we found that lncRNA TUG1 was mainly localized in the cytoplasm (Figure 2e).

### LncRNA TUG1 negatively regulates miR-338 expression via sequestering

**Figure 3.**
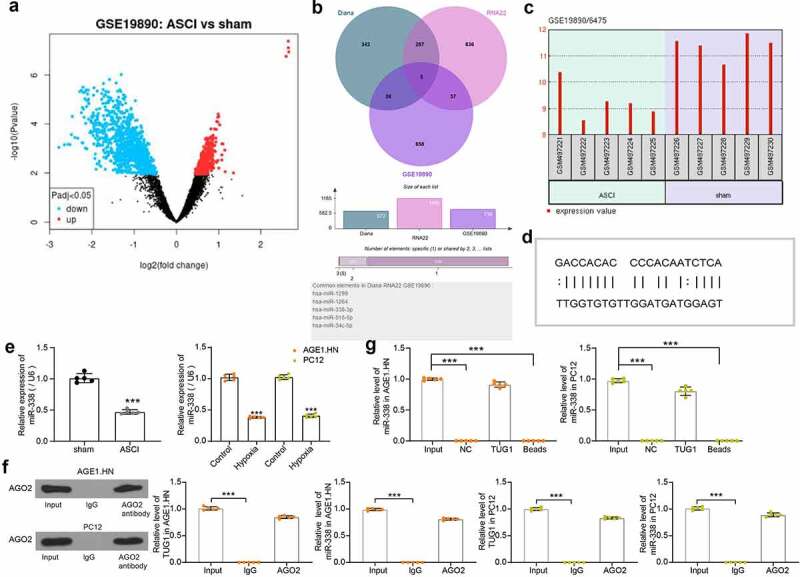
TUG1 downregulates miR-338 expression in AGE1.HN and PC-12 cells. (a) screening of differentially expressed miRNAs in spinal cord tissue of SCI rats by GEO database. (b) the intersection of potential binding miRNAs for TUG1 and downregulated miRNAs in the GEO database. (c) expression of miR-338 in spinal cord tissues of SCI rats as queried in GEO database. (d) the potential binding relationship between TUG1 and miR-338 predicted by RNA22. (e) miR-338 expression in rat tissues (n = 5) and in AGE1.HN and PC12 cells treated with hypoxia (unpaired *t* test, ***p < 0.001; rat: t(8) = 15.067, p < 0.001: AGE1.HN: t(4.693) = 27.339, p < 0.001; PC12: t(8) = 29.803, p < 0.001). (f) AGO2 antibody precipitate was enriched in TUG1 and miR-338 verified by RIP normalized to IgG (one-way ANOVA, ***p < 0.001. AGE1.HN: F(2,12) = 2698.390, p < 0.001, F(2,12) = 5214.375, p < 0.001; PC12: F(2,12) = 3525.508, p < 0.001, F(2,12) = 1959.624, p < 0.001). (g) the enrichment of miR-338 in PC-12 cells by TUG1 determined by RNA pull-down normalized to IgG (one-way ANOVA, ***p < 0.001. AGE1.HN: F(3,16) = 2787.589, p < 0.001; PC12: F(3,16) = 956.353, p < 0.001). Data were displayed as the mean ± SD. Five independent assays were conducted only for *in vitro* experiments

To search for downstream miRNAs through which TUG1 plays a role in ASCI, we first selected the dataset GSE19890 in the GEO database to screen out miRNAs that are differentially expressed in the spinal cord tissues of SCI rats (Figure 3a). Subsequently, we predicted miRNAs with binding relationship with TUG1 in Diana (https://carolina.imis.athena-innovation.gr/diana_tools/web/) and RNA22 (https://cm.jefferson.edu/rna22/), respectively, followed by intersection with miRNAs significantly reduced in GSE19890. We screened five intersecting miRNAs (Figure 3b). Among them, only miR-338 was reported to play a key role in SCI [29,30] and was therefore selected for further study. With the GSE19890 dataset, we confirmed the poor expression of miR-338 (GPL9908 platform number: 6475) in SCI (Figure 3c). We then downloaded the potential binding sites between TUG1 and miR-338 in the bioinformatics website RNA22 (Figure 3d). miR-338 was revealed to be reduced in tissues of the rats with SCI relative to that in sham-operated rats. Likewise, miR-338 was downregulated in hypoxia-treated AGE1.HN and PC12 cells versus normoxic AGE1.HN and PC12 cells (Figure 3e).

AGO2 involved in the miRNA assembly process is the key component of the RNA induced silencing complex [31]. RIP was then performed in AGE1.HN and PC12 cell extracts using an antibody to AGO2. By western blot and RT-qPCR, we found that there was no significant difference in the levels of TUG1 and miR-338 that could be enriched using AGO2 antibody compared to Input, while IgG antibody enriched less TUG1 and miR-338 in AGE1.HN and PC12 cells (figure 3f). We also performed RNA pull-down with biotinylated lncRNA TUG1 in AGE1.HN and PC12 cells (Figure 3g). The miR-338 expression in the pull-down complexes was analyzed by RT-qPCR. miR-338 expression detected in the complexes pulled-down by TUG1 was not significantly different compared to Input, while NC or blank magnetic beads were barely enriched for miR-338. It was thus proved that lncRNA TUG1 bound specifically to miR-338.

### LncRNA TUG1 inhibits miR-338 expression to promote AGE1.HN and PC12 death

**Figure 4.**
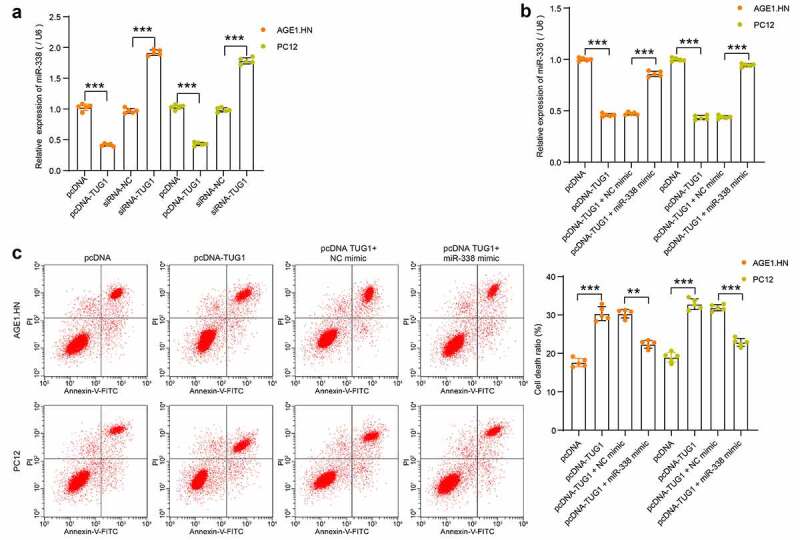
TUG1 inhibits miR-338 expression to promote AGE1.HN and PC12 cell death. (a) miR-338 expression in AGE1.HN and PC-12 cells in response to pcDNA-TUG1 or siRNA-TUG1 determined by RT-qPCR normalized to GAPDH (one-way ANOVA, ***p < 0.001. AGE1.HN: F(3,16) = 1143.706, p < 0.001; PC12: F(3,16) = 1053.589, p < 0.001). (b) miR-338 expression in AGE1.HN and PC-12 cells in response to pcDNA-TUG1 or pcDNA-TUG1 + miR-338 mimic determined by RT-qPCR normalized to GAPDH (one-way ANOVA, ***p < 0.001. AGE1.HN: F(3,16) = 1168.594, p < 0.001; PC12: F(3,16) = 1667.774, p < 0.001). (c) cell death in AGE1.HN and PC-12 cells in response to pcDNA-TUG1 or pcDNA-TUG1 + miR-338 mimic determined by flow cytometry (one-way ANOVA, **P = 0.002, ***p < 0.001. AGE1.HN: F(3,16) = 117.167, p < 0.001; PC12: F(3,16) = 173.777, p < 0.001). Data were displayed as the mean ± SD. Five independent assays were conducted only for *in vitro* experiments

To confirm the binding relationship, human or rat pcDNA-TUG1, siRNA-TUG1 and NCs were transfected into hypoxia-treated AGE1.HN and PC12 cells, respectively. miR-338 expression was measured with RT-qPCR. It was found that pcDNA-TUG1 significantly repressed miR-338 expression, while siRNA-TUG1 enhanced its expression in hypoxia-treated AGE1.HN and PC12 cells (Figure 4a). miR-338 mimic and NC mimic were then transfected into AGE1.HN and PC12 cells overexpressing lncRNA TUG1. RT-qPCR data illustrated that miR-338 expression, suppressed by lncRNA TUG1, was enhanced by miR-338 mimic in AGE1.HN and PC12 cells (Figure 4b). Flow cytometry was then applied on AGE1.HN and PC12 cells after co-transfection. miR-338 mimic was observed to flatten the stimulative role of lncRNA TUG1 on AGE1.HN and PC12 cell death (Figure 4c). The experimental results showed that lncRNA TUG1 promoted AGE1.HN and PC12 cell death under hypoxia environment by downregulating miR-338 expression.

### BIK is a downstream target of miR-338

**Figure 5.**
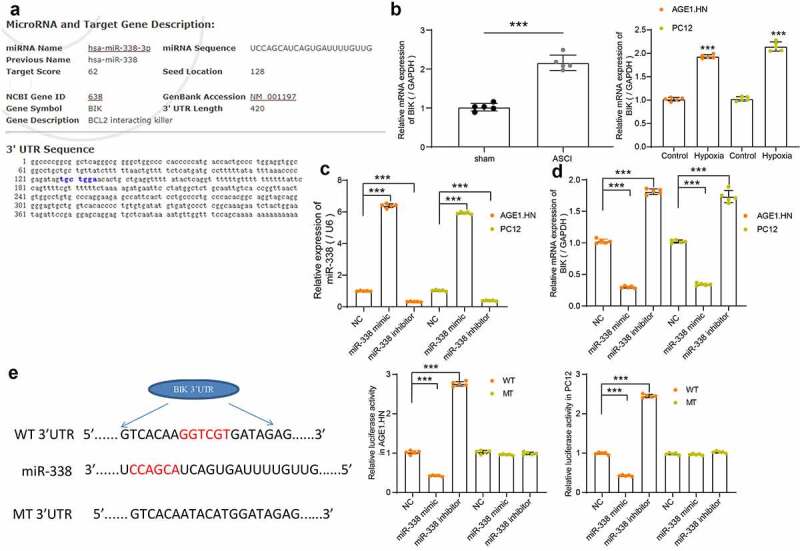
BIK is a possible target of miR-338 in AGE1.HN and PC12 cells. (a) the potential binding sites between the 3ʹUTR of BIK and miR-338. (b) mRNA expression of BIK in spinal cord tissues of sham-operated (n = 5) and ASCI rats (n = 5) and in AGE1.HN and PC12 cells treated with or without hypoxia were measured by RT-qPCR normalized to GAPDH (unpaired *t* test, ***p < 0.001, rat: t(8) = −11.587, p < 0.001; AGE1.HN: t(8) = −37.693, p < 0.001; PC12: t(8) = −22.900, p < 0.001). AGE1.HN and PC12 cells were delivered with miR-338 mimic or inhibitor. (c) the miR-338 expression in AGE1.HN and PC-12 cells determined by RT-qPCR normalized to U6 (one-way ANOVA, ***p < 0.001. AGE1.HN: F(2,12) = 10,440.468, p < 0.001; PC12: F(2,12) = 18,635.483, p < 0.001). (d) the BIK mRNA expression in AGE1.HN and PC-12 cells determined by RT-qPCR (one-way ANOVA, AGE1.HN: F(2,12) = 2552.089, p < 0.001; PC12: F(2,12) = 697.459, p < 0.001). (e) the relative luciferase activity was tested by dual-luciferase reporter assays after co-transfection with miR-338 mimic/inhibitor or NC and BIK-WT or BIK-MT (one-way ANOVA, ***p < 0.001. AGE1.HN: WT F(2,12) = 4049.642, p < 0.001, MT F(2,12) = 5.411, p = 0.021; PC12: WT F(2,12) = 6070.952, p < 0.001, MT F(2,12) = 9.165, p = 0.004). Data were displayed as the mean ± SD. Five independent assays were conducted only for *in vitro* experiments

The potential binding sites between miR-338 and BIK were predicted by Starbase, which were located on the conserved sequences of rat and human genes (Figure 5a). BIK was revealed to be elevated in tissues of rats with SCI relative to that in sham-operated rats. Likewise, BIK was upregulated in hypoxia-treated AGE1.HN and PC12 cells versus normoxic AGE1.HN and PC12 cells (Figure 5b). To investigate the correlation between miR-338 and BIK, AGE1.HN and PC12 cells were delivered with human or rat miR-338 mimic/inhibitor and their NCs. Following the determination of successful transfection in AGE1.HN and PC12 cells (Figure 5c), BIK mRNA expression was measured using RT-qPCR. We found that miR-338 mimic inhibited the expression of BIK, while miR-338 inhibitor increased that of BIK in AGE1.HN and PC12 cells (Figure 5d). Dual-luciferase reporter assay displayed that the relative luciferase activity of vector containing BIK-WT in AGE1.HN and PC12 cells was distinctively diminished when co-transfected with miR-338 mimic and enhanced when co-transfected with miR-338 inhibitor (Figure 5e). Collectively, miR-338 negatively regulated BIK expression.

### BIK promotes AGE1.HN and PC12 cell death

**Figure 6.**
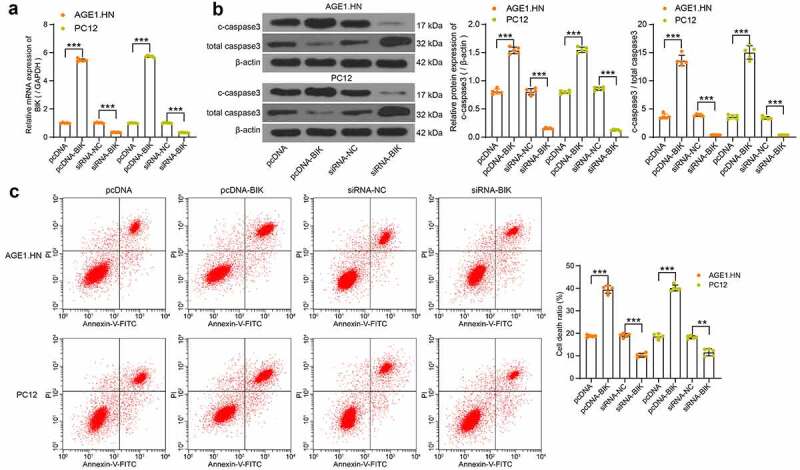
BIK facilitates AGE1.HN and PC12 cell death. AGE1.HN and PC12 cells were delivered with pcDNA-BIK or siRNA-BIK. (a) the BIK mRNA expression in AGE1.HN and PC-12 cells determined by RT-qPCR (one-way ANOVA, ***p < 0.001. AGE1.HN: F(3,16) = 9423.939, p < 0.001; PC12: F(3,16) = 19,510.157, p < 0.001). (b) the protein expression of c-caspase3 and c-caspase3/total caspase3 in AGE1.HN and PC12 cells after transfection measured by western blot analysis normalized to β-actin (one-way ANOVA, ***p < 0.01. For c-caspase3: AGE1.HN: F(3,16) = 750.932, p < 0.001; PC12: F(3,16) = 1644.487, p < 0.001. For c-caspase3/total caspase3: AGE1.HN: F(3,16) = 583.2, p < 0.001; PC12: F(3,16) = 513.6, p < 0.001). (c) the death rate of AGE1.HN and PC12 cells after transfection measured by flow cytometry (one-way ANOVA, **p = 0.002, ***p < 0.001. AGE1.HN: F(3,16) = 648.677, p < 0.001; PC12: F(3,16) = 517.799, p < 0.001). Data were displayed as the mean ± SD. Five independent assays were conducted only for *in vitro* experiments

Human or rat pcDNA-BIK, siRNA-BIK and their respective NCs were transfected into hypoxia-treated AGE1.HN and PC12 cells, RT-qPCR confirmed the successful transfection (Figure 6a). Western blot analysis and flow cytometry illustrated that pcDNA-BIK notably increased c-caspase3 expression and c-caspase3/total caspase3 in AGE1.HN and PC12 cells (Figure 6b) and death of AGE1.HN and PC12 cells (Figure 6c), while siRNA-BIK reduced these trends. All in all, it was found that BIK plays a role in promoting AGE1.HN and PC12 cell death.

***Knockdown of lncRNA TUG1 alleviates SCI by inhibiting death of AGE1.HN and PC12 cells via the miR-338/BIK axis***

**Figure 7.**
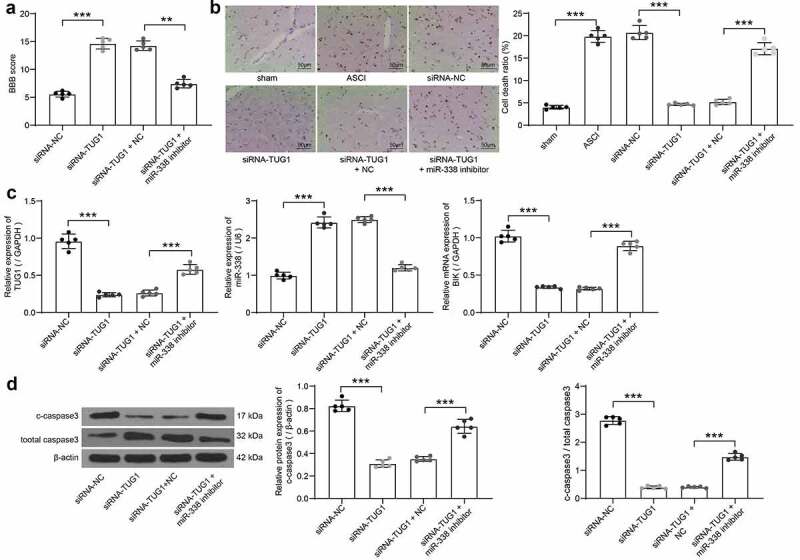
The regulatory role of TUG1/miR-338/BIK axis on rats with SCI (n = 5). (a) the BBB scores for hindlimb locomotion in the rats with ASCI injected with siRNA-TUG1 alone or with miR-338 inhibitor (one-way ANOVA, **p = 0.008, ***p < 0.001. F(3,16) = 175.553, p < 0.001). (b) detection of cell death in rat spinal cord by TUNEL staining (one-way ANOVA, ***p < 0.001. F(5,24) = 296.154, p < 0.001). (c) TUG1 (normalized to GAPDH) and miR-338 expression (normalized to U6) as well as the BIK mRNA expression (normalized to GAPDH) in spinal cord tissues of rats determined by RT-qPCR (one-way ANOVA, ***p < 0.001. TUG1: F(3,16) = 140.714, p < 0.001; miR-338: F(3,16) = 291.155, p < 0.001; BIK: F(3,16) = 251.190, p < 0.001). (d) the protein expression of c-caspase3 and c-caspase3/total caspase3 in spinal cord tissues of rats measured by western blot analysis normalized to β-actin (one-way ANOVA, ***p < 0.001. For c-caspase3/total caspase3: F(3,16) = 146.102, p < 0.001. For c-caspase3/total caspase3: F(3,16) = 702.9, p < 0.001). Data were displayed as the mean ± SD. Five independent assays were conducted only for *in vitro* experiments

To verify the role of lncRNA TUG1/miR-338/BIK axis in SCI, we injected siRNA-NC, siRNA-TUG1, siRNA-TUG1 + NC or siRNA-TUG1 + miR-338 inhibitor into the ASCI rats intrathecally, with five rats in each group. The hindlimb movement of rats was observed after 15 days. siRNA-NC rats had only slight movement of the hindlimb joints, showing severer dyskinesia. There was no significant difference between rats injected with siRNA-TUG1 and siRNA-TUG1 + NC. Rats in both groups exhibited consistent plantar stepping and consistent coordination of hindlimb and forelimb; and no toe clearance or occasional toe clearance during forward limb advancement. The hindlimbs of siRNA-TUG1 + miR-338 inhibitor-treated rats showed extensive movement, but the hindlimbs were not coordinated with the forelimbs. The BBB score evaluation on hindlimb locomotor activity in rats revealed that knockdown of lncRNA TUG1 significantly enhanced the score, while the score was decreased after miR-338 inhibitor, which was still higher than that of siRNA-NC-treated rats (Figure 7a). It was suggested that inhibition of miR-338 partially abrogated the attenuating effect of lncRNA TUG1 knockdown on SCI.

The spinal cord tissues were collected, and the cell death in the spinal cord tissues was detected by TUNEL staining. ASCI modeling led to increased death rates in spinal cord tissues. The cell death was the highest in the siRNA-NC and the model groups, followed by the siRNA-TUG1 + miR-338 group, and that of the siRNA-TUG1 and siRNA-TUG1 + NC groups were relatively low (Figure 7b). RT-qPCR was used to evaluate the expression of lncRNA TUG1, BIK and miR-338 in tissues of rats after delivery. Knockdown of lncRNA TUG1 significantly reduced the expression of BIK and lncRNA TUG1, and promoted the expression of miR-338, which was partially reversed after miR-338 inhibitor (Figure 7c). Finally, western blot was utilized to measure the c-caspase3 expression and c-caspase3/total caspase3 in the tissues. LncRNA TUG1 knockdown notably repressed the expression of c-caspase3 and c-caspase3/total caspase3, but miR-338 inhibitor weakened this repression (Figure 7d). These results hinted that lncRNA TUG1 knockdown repressed the expression of BIK by targeting miR-338, thus inhibiting AGE1.HN and PC12 cell death and improving SCI.

## Discussion

Traumatic SCI could be divided into three phases, an acute phase, a secondary phase and a chronic phase that characterized by apoptosis, Wallerian degeneration as well as scarring which establishes functional impairment [32]. Our findings suggested that lncRNA TUG1 and BIK were significantly upregulated, whereas miR-338 was diminished in the spinal cord tissues of SCI rats. In addition, lncRNA TUG1 inhibition promoted hindlimb locomotor activity in ASCI rats and halted cell death as evidenced by diminished c-caspase3. Also, it was revealed that lncRNA TUG1 knockdown exerted an anti-apoptotic role by binding to miR-338, thus decreasing the expression BIK in ASCI rats and AGE1.HN and PC12 cells.

Initially, lncRNA TUG1 was found expressed highly in spinal cord tissues and AGE1.HN and PC12 cells. Moreover, lncRNA TUG1 overexpression enhanced the death of these cells by elevating the c-caspase3 expression and c-caspase3/total caspase3. By contrast, lncRNA TUG1 knockdown by siRNAs led to the opposite trends. Aberration in lncRNA expression of murine models following traumatic or nontraumatic SCI has been revealed by gene expression profiles, and lncRNAs are tightly correlated with numerous pathophysiological events, including inflammatory response and neuron loss [33]. For instance, ANRIL worsened hydrogen peroxide-evoked injury in PC-12 cells by suppressing miR-499a/PDCD4 axis [34]. LncRNA TUG1, a 7.1-kb lncRNA, precipitates in numerous biological events through chromatin remodeling or performing as decoys for proteins or miRNAs [35]. For example, lncRNA TUG1 suppressed miR-132-3p expression via direct interaction, and delivery of miR-132-3p inhibitor partially flattened the effect of lncRNA TUG1 downregulation on the apoptosis of osteosarcoma cells [36]. For its individual role, lncRNA TUG1 promoted glioma cell apoptosis by activating caspase3-dependent intrinsic pathway, performing as a tumor suppressor in glioma [37]. Moreover, lncRNA TUG1 enhanced apoptosis of lens epithelial cells by modulating the miR-421/caspase-3 network in age-related cataract [38]. On the contrary, silencing of lncRNA TUG1 protected human nucleus pulposus cells from apoptosis and senescence evoked by TNF-α by diminishing caspase-3 expression [39]. Rat hippocampal neurons treated by si-TUG1 + Ketamine exhibited lower levels of TUG1 and c-caspase3 than those treated by Ketamine alone [40]. Considering the aforementioned findings, we concluded that lncRNA TUG1 is closely linked to death of cells. Likewise, TUNEL staining on cells extracted from spinal cord tissues of ASCI rats uncovered that lncRNA TUG1 knockdown reduced the cell death *in vivo*.

In order to study the regulatory mechanism, we next concentrated on the downstream miRNAs of lncRNA TUG1. Bioinformatics analysis unearthed that lncRNA TUG1 could bind to the sequence of miR-338. miR-338-5p upregulation has been verified to suppress the apoptosis of Neuro-2a cells exposed to hypoxia/reoxygenation, thus alleviating cerebral ischemia/reperfusion injury [41]. Besides, we also found that miR-338 mimic could flatten the effects of lncRNA TUG1 on AGE1.HN and PC12 cell death under hypoxia treatment, which substantiated that downregulation of miR-338 was a reason for the stimulative effects of lncRNA TUG1 on AGE1.HN and PC12 cell death. Moreover, MALAT1 induced the expression of hypersensitive C-reactive protein by repressing the expression of hsa-miR-338-3p to promote acute cerebral infarction [42]. Furthermore, circ_0001313 downregulation increased caspase-3 expression in colon cancer cells after irradiation, whereas miR-338-3p reversed the functions of circ_0001313 downregulation on caspase-3 activity [43]. The current work displayed that lncRNA TUG1 overexpression significantly enhanced c-caspase3 activity, while miR-338 restoration reduced the c-caspase3 expression in the presence of lncRNA TUG1 overexpression. In addition, the dual-luciferase activity assay revealed that BIK was a downstream target gene of miR-338, and miR-338 could conversely modulate BIK expression. The major form of cell death is apoptosis, which is a programmed event regulated by the BCL-2 family of proteins which can be divided into two groups: the effectors and the BH3-only proteins, including BIK [44]. Previously, miR-1306-5p overexpression has been indicated to alleviate injury caused by cerebral ischemia/reperfusion *in vitro* by targeting BIK [45]. Moreover, BIK has been reported to accelerate influenza A infection by promoting the induction of caspase3 and cytoplasmic export of viral RNPs [46]. In the current work, we found that BIK overexpression promoted the activation of c-caspase3, while BIK downregulation lowered its expression, thus prohibiting the cell death. However, the exact mechanism underlying BIK-mediated neuronal apoptosis has not been fully elucidated in the present study. Therefore, further experiments are warranted on this perspective.

## Conclusion

**Figure 8.**
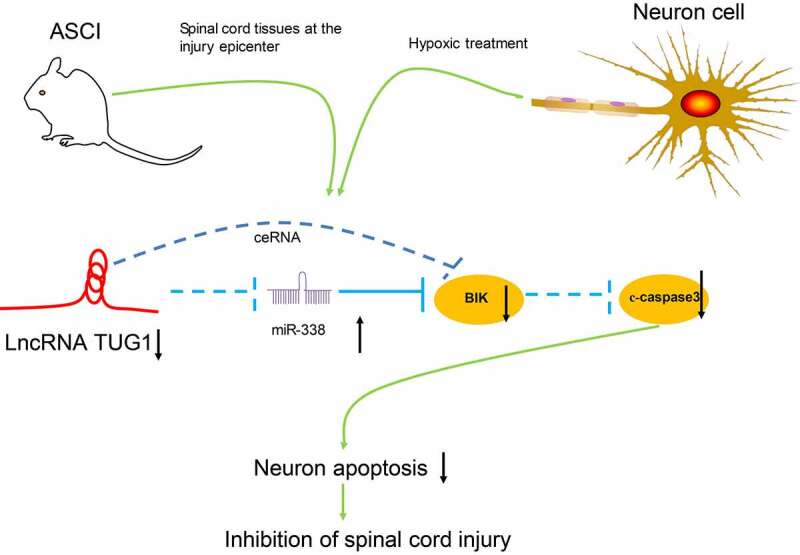
Mechanisms of TUG1 mediated SCI progression. TUG1 interacts with miR-338, leading to BIK activation, which accelerates SCI via promoting cell death. While TUG1 knockdown downregulates BIK expression by interacting with miR-338, thus lowering c-caspase3 to hamper death

Taken together, our present experiments illustrated that lncRNA TUG1 knockdown protected AGE1.HN and PC12 cells against death. The neuroprotective effects of lncRNA TUG1 knockdown *in vitro* and in SCI rats may be achieved via upregulating miR-338 and downregulating BIK. After the delivery of siRNA-TUG1 into the AGE1.HN and PC12 cells, the expression of miR-338 is altered and the following neuronal death is inhibited, which ultimately ameliorates SCI (Figure 8). Therefore, this study expands our understanding of the lncRNA-miRNA-mRNA regulation network associated with SCI and may supply a new therapeutic agent aimed at reducing neuronal injury in spinal cord tissues after SCI. Nevertheless, for time and funding constraints, we did not investigate other mechanisms of cell death (e.g., pyroptosis and autophagy), which remains to be explored in our future work.

## Data Availability

The data used to support the findings of this study are available from the corresponding author upon request.

## Highlights

1. High expression of TUG1 in SCI mice and AGE1.HN and PC12 under hypoxia.
2. TUG1 and BIK promote apoptosis of AGE1.HN and PC12 cells.
3. There is a binding relationship between TUG1 and miR-338.
4. miR-338 targets and negatively regulates BIK expression.
5. TUG1 regulates BIK expression through competitive binding to miR-338.
